# Reduced positive attentional bias in patients with borderline personality disorder compared with non-patients: results from a free-viewing eye-tracking study

**DOI:** 10.1186/s40479-024-00267-y

**Published:** 2024-09-16

**Authors:** Taavi Wenk, Anna-Christina Günther, Carolin Webelhorst, Anette Kersting, Charlott Maria Bodenschatz, Thomas Suslow

**Affiliations:** Department of Psychosomatic Medicine and Psychotherapy, University of Leipzig Medical Center, Semmelweisstr. 10, 04103 Leipzig, Germany

**Keywords:** Attentional bias, Borderline personality disorder, Eye movements, Facial emotions, Happy faces, Positivity bias

## Abstract

**Background:**

Attentional processes are important for regulating emotional states and coping with stressful events. Orientation of attention acts as filter for subsequent information processing. So far, only few eye-tracking studies have examined attentional processes during emotion perception in borderline personality disorder (BPD). In these studies, gaze behaviour was analysed during simultaneous or delayed evaluation of single stimuli. The objective of the present eye-tracking study was to investigate early and late attention allocation towards emotional facial expressions in patients with BPD and non-patients (NPs) based on a free-viewing paradigm, which allows to examine processes of self-generated attention deployment.

**Methods:**

In a multiple-stimulus free-viewing task with facial expressions, i.e. happy, angry, sad, and neutral faces, presented simultaneously early and late attentional allocation was analysed in 43 patients with BPD and 43 age- and sex-matched NPs. We assessed study participants’ trait anxiety, depressive symptoms, level of alexithymia, traumatic childhood experiences, and borderline symptoms. Entry time was used to measure initial gaze orientation, whereas dwell time was calculated as an index of late attention allocation.

**Results:**

As could be expected, patients with BPD reported more anxiety, depressive symptoms, experiences of childhood maltreatment, and showed higher levels of alexithymia than NPs. Patients differed from NPs in dwell time on happy facial expressions but not in dwell time on angry, sad, and neutral expressions. Contrary to our hypothesis, patients did not differ from NPs concerning entry times on angry facial expressions.

**Conclusions:**

According to our results, patients with BPD show a reduced attentional preference for happy facial expression during free viewing compared to NPs. A decreased positive attentional bias at a late processing stage could be part of emotion regulation impairments and add to the vulnerability for negative affects in BPD, which represent core symptoms of the disorder. In contrast to previous eye-tracking research in BPD examining attention during evaluative processing, our dwell time data could be more indicative of self-generated, endogenously controlled attentional processes in emotion perception. The present data do not support an early vigilance for threatening social information in BPD.

**Supplementary Information:**

The online version contains supplementary material available at 10.1186/s40479-024-00267-y.

## Background

Borderline personality disorder (BPD) is a severe mental disorder characterized by emotional and interpersonal instability, impulsive and self-damaging behaviours [1]. It occurs with a considerable lifetime prevalence of up to 5.5% [2]. Linehan’s biosocial theory of BPD postulates that the interaction between a child’s biologically based vulnerability and invalidating environmental responses results in a pervasive pattern of emotion dysregulation [3]. This theory delineates three core aspects of emotional responding in BPD: higher emotional sensitivity and emotional reactivity, and a slow return to baseline [3].

Patients with BPD often utilise maladaptive emotion regulation strategies, including avoidance [4], thought suppression [5], and rumination [6]. BPD is also associated with low stress tolerance [7]. Accordingly, negative affect is a core feature of BPD [8, 9]. Emotion dysregulation is commonly regarded as one of the main features of BPD [10]. An important component of emotion regulation is the regulation of attention [11, 12]. As attentional processes have been identified as a potential causal factor in the development and maintenance of emotional disorders such as panic disorder, depression, anxiety or post-traumatic stress disorder [13], it is important to gain an in-depth understanding of attentional alterations in BPD. As attention allocation acts as a filter for subsequent information processing [14], it can, for example, enhance reward perception or result in negative emotional responses, such as distress [15]. Recent research suggests that attentional biases may play an important role in BPD (e.g [16, 17]). As there exists a close coupling of gaze direction and attention allocation [18], eye-tracking has become a widely used method of measuring explicit attention to visual stimuli. Eye-tracking research allows assessing early as well as late attentional processes (e.g [19]). Early gaze behaviour indicates processes of initial attention orientation whereas subsequent gaze behaviour allows to assess attentional maintenance or preference [20].

### Research on early attention allocation in BPD

When non-patients (NPs) look at emotional images presented together with neutral ones, their initial orientation of attention is biased towards the emotional images regardless of valence [20, 21]. According to Linehan’s biosocial model, patients with BPD exhibit heightened sensitivity to threat-related stimuli [3]. This assumption has been corroborated by several studies [22, 23]. Thus, it appears that BPD patients may direct their attention towards threatening information during early stages of processing and that they could differ from non-patients in their initial attention orientation and manifest *early threat vigilance* (see also [24]). This is suggested by studies utilizing the dot-probe and emotional Stroop paradigm [16, 17]. However, there has been little eye-tracking research examining early attentional processes in BPD. In the study of Bertsch and colleagues [25], an emotion classification paradigm was employed to investigate the gaze patterns on single faces of individuals with BPD compared to those without the disorder. Their results indicate that BPD patients make faster initial fixation changes towards the eyes of angry and fearful faces than non-patients. A subsequent study using the same paradigm found that patients with BPD make faster initial fixation changes towards the eye region of neutral faces, whereas no effect was found for angry faces [26]. Furthermore, Seitz et al. [27] observed faster and more fixation changes towards the eyes of emotional and neutral faces in BPD compared to healthy individuals in an emotion classification paradigm. These findings are coherent with the view that BPD patients are hypersensitive to threat in early stages of information processing. In an experiment in which positive, negative and neutral socio-emotional pictures were presented individually, and participants had the task to rate the valence of the images, Bortolla and colleagues [19] observed no differences in first fixation latency between BPD patients and healthy controls. In a subsequent study, the first fixation latency was increased in BPD patients for negative socio-emotional content when compared to healthy controls, which may indicate an early avoidance of negative social information in BPD [28]. Given the inconsistencies of findings in the aforementioned studies, further research is required to determine whether BPD is associated with an early vigilance or an early avoidance of negative emotional content. To the best of our knowledge, no eye-tracking study has yet been conducted examining the early allocation of attention to simultaneously presented multiple facial expressions.

### Eye-tracking research on late attention allocation in BPD

When non-patients view at simultaneously presented emotional and neutral images they look longer at the emotional images irrespective of their valence [21]. Late attention allocation to negative facial expressions has been observed to be related to negative affective states such as depressive mood [29], whereas a bias in attention allocation to positive content is found in healthy individuals [30]. A positive attentional bias at late processing stages is assumed to have mood stabilizing or mood enhancing functions and can be interpreted as a form of emotion regulation [31, 32]. Typically, clinically depressed patients exhibit a decreased positive attentional bias (e.g [33], see [34] for a meta-analytic review).

There is some evidence supporting the hypothesis that BPD is associated with an increased attention allocation to negative emotional content [16, 17]. However, other findings suggest avoidance tendencies for fearful and happy facial expressions in BPD (*late threat avoidance*; [35]). Recent eye-tracking results indicate that patients with BPD explore positive and negative socio-emotional content less compared to healthy individuals [19]. Similarly, in a subsequent eye-tracking study, Bortolla and colleagues [28] observed that patients with BPD spend less time exploring negative and neutral socio-emotional scenes. Against the background of recent findings, it could be concluded that avoidance of social emotional information might characterize BPD patients at late stages of attention allocation. Therefore, despite some inconsistencies, existing eye-tracking research provides some evidence for both, a decreased attentional bias towards positive content and avoidance of negative content in BPD. An influential variable in eye-tracking research on attentional processes is the experimental task instruction [21]. In a recent study [36], simultaneous fMRI and eye-tracking measurements during an emotional face matching task containing happy, neutral, sad, angry and fearful facial expression were used to assess emotional attentional biases in clinically depressed patients and NPs. The authors compared gaze behaviour during emotion recognition with gaze behaviour during free-viewing based on the same stimulus material and observed a mood congruent pattern in depressed patients only in the free-viewing condition. Interestingly, the fMRI results showed that when contrasting free viewing vs. emotion recognition (based on data of attentional preference for emotional faces), free viewing was more strongly associated with activity in the dorsolateral prefrontal cortex, whereas emotion recognition was linked to greater activation of the primary visual cortex. The authors concluded that identification of emotions leads to a more feature-based visual processing while free-viewing involves more spontaneous attentional responses depending on an individual’s self-referential schemes and mood state, which could be indicative of a more endogenous control compared to task-related gaze behaviour. Previous eye-tracking research with BPD patients examined gaze behaviour during simultaneous or delayed evaluation of socio-emotional pictures [19, 28] or emotion classification of facial expressions [25–27]. Thus, it can be assumed that in these studies gaze behaviour was analysed, which at least in part was task-related. Until now, there is no eye-tracking study examining processes of late attention allocation in BPD using a free-viewing paradigm with facial expressions and no additional task. The multi-stimulus free-viewing task using facial, pictorial, or lexical stimuli constitutes an often applied paradigm in eye-tracking research that asks participants to observe images freely without constraints on attention [37]. Free-viewing tasks provide estimates of early and late processes of spontaneous attention allocation, e.g., indices of initial orienting to or sustained attention on specific stimulus categories [38]. In the last decades, studies based on the free-viewing task have substantially contributed to reveal anxiety- and depression-related attentional alterations [34, 39, 40]. The free-viewing paradigm has proven to be reliable across multiple measurements, different types of stimuli, and participant groups [41]. Moreover, recent research demonstrated moderate to excellent internal consistency for the free-viewing paradigm and adequate to good test-retest reliability for attentional biases regarding dwell time [42]. The application of a free-viewing paradigm thus provides a reliable means of studying processes of self-generated attention deployment.

### The present study

The main aim of the study was to determine how patients with BPD differ from NPs concerning early and late attention allocation to facial expressions. We assessed gaze behaviour during a free-viewing task in which four categories of facial expressions (i.e., happiness, anger, sadness and neutral) were shown simultaneously. Firstly, we expected that patients with BPD exhibit faster initial attention allocation towards angry facial expressions (indicating an *early threat vigilance).* Because it is known that pictures with emotional content are looked at first (e.g [21]), we additionally analysed entry times as a function of facial expression categories. Secondly, we hypothesised that patients with BPD spend less time fixating on happy faces (indicating a decreased *positive attentional bias*). A subordinate aim of this study was to identify indicators for *late threat avoidance*. Because it has been shown that patients with BPD exhibit attentional avoidance concerning negative socio-emotional content and fearful facial expressions [19, 28, 35], we assumed that patients with BPD also spend less time fixating on angry faces.

Recent research demonstrated that attentional processes towards emotional content depend on levels of childhood maltreatment (CM; [43]) and alexithymia [44] of individuals. Furthermore, anxiety [45, 46] and depression [34, 47] were found to be associated with alterations in attentional processes towards emotional content. In the context of attentional processes in BPD, the role of these variables is not yet fully understood. We therefore decided to investigate whether depression, anxiety, CM, and alexithymia are related to late attention allocation. Since comorbidities are high amongst individuals with BPD [48] and recent eye-tracking research on patients with BPD did not exclude axis I comorbidities (e.g [19, 25–28]) we decided to include patients with comorbid axis I disorders.

## Methods

### Participants

All participants in this study were aged between 18 and 45 years and were native speakers of German. The procedure of the study was explained before the experiment. All participants were financially compensated upon completion of the study. The general exclusion criteria for all participants were: (1) current or lifetime neurological disorder; (2) head injury with a possible negative impact on cognitive function; (3) current substance dependence or substance abuse; (4) drug use on the day of experiment; (5) current medication of benzodiazepines; and (6) compromised vision. Visual acuity was assessed using the *Snellen eye chart*. All other exclusion criteria were assessed by self-report.

As evidenced by prior research on depression employing the free-viewing paradigm, a minimum of 31 individuals per group is required to detect group differences with sufficient statistical power (see [47]). Therefore, we decided to attain a group size of 50 individuals per group. BPD patients were recruited from the Department of Psychosomatic Medicine and Psychotherapy at the University of Leipzig. The patients with BPD (who were interested in our study) were referred to us by the senior physician of the department. BPD patients and NPs were tested with the Structured Clinical Interview for the DSM-IV Axis I and Axis II (SCID-I and SCID-II German version [49]), by two trained interviewers (a clinical psychologist or a medical doctoral student) to determine their study eligibility. Exclusion criteria for BPD patients were a diagnosis of bipolar disorder, psychotic disorder, or schizoaffective disorder. Initially, 51 patients with BPD were included in our study. After the diagnostic interview, four patients who did not achieve a minimum score of five out of nine DSM-IV BPD criteria were excluded. Two patients who were currently abusing substances were also excluded from the study. Two other patients were unable to participate in the experiment due to the impact of the pandemic situation of COVID-19. The final sample consisted of 43 BPD patients (36 female) and 43 non-patients. We matched the groups on an individual level for age and biological sex. The SCID-II also indicated the presence of several comorbid personality disorders in the BPD group. Comorbid axis I and axis II disorders and medication intake in the BPD patient group is shown in Table 1.

Participants for the NP group were recruited via online advertisements and public notices posted in the city. The exclusion criteria for the NP group were: (1) current or lifetime diagnosis of any psychiatric disorder; (2) past or current use of psychotherapy; (3) minimal, moderate, or severe depression (BDI-II ≥ 9); and (4) exceeding the cut-off score for moderate BPD symptoms (BSL-23 score > 1.07 [50]). The NP group was screened using the SCID-I and SCID-II to exclude the presence of mental and personality disorders. Descriptive statistics of sociodemographic and psychological variables for both groups are shown in Table 2.

**Table 1:** Comorbid axis I and axis II disorders and medication intake in the BPD group (*n* = 43)

**Comorbid axis-I disorder**	***n***	
Affective disorder	25	
Anxiety disorder	22	
PTSD	12	
Eating disorder	18	
Somatoform Disorder	7	
**Comorbid personality disorder**	*n*	
Obsessive compulsive	19	
Depressive	18	
Avoidant	14	
Dependent	8	
Antisocial	7	
Paranoid	7	
Narcissistic	5	
Schizotypal	3	
Without a specified diagnosis	8	
**Medication intake**	*n*	
SSRI	11	
SNRI	3	
SDRI	1	
TeCA	1	
TCA	2	

Note: SSRI = Selective Serotonin Reuptake Inhibitor; SNRI = Serotonin-Noradrenalin-Reuptake-Inhibitor; SDRI = Serotonin–dopamine reuptake inhibitor; TeCA = Tetracyclic antidepressant; TCA = Tricyclic antidepressant

### Measures and materials

In order to assess the specific symptoms associated with borderline personality disorder, the *Borderline Symptom List* (BSL-23; [51]) was administered to all participants. It is based on the DSM-IV and consists of 23 items asking participants to rate how much they have experienced each symptom of BPD over the previous week on a 5-point Likert scale. According to Kleindienst et al. [50], a mean of 1.07 or above indicates moderate severity, and a mean of 1.87 or above indicates high severity. The internal consistency of the BSL-23 in the current sample was very good (α = 0.903).

The *Beck Depression Inventory* (BDI-II; German Version: [52]) was administered to ascertain the severity of depressive symptoms. The 21-item self-report questionnaire assesses symptoms such as negative cognitions, hopelessness, and physical symptoms during the preceding two weeks. The BDI-II total score can range from 0 to 63, with higher scores indicating more severe symptoms. The internal consistency of the BDI-II in the current sample was excellent (α = 0.945).

All participants completed the *Childhood Trauma Questionnaire* (CTQ; German version: [53]). The CTQ is a retrospective self-report questionnaire consisting of five subscales (emotional abuse, physical abuse, sexual abuse, emotional neglect, and physical neglect). Each subscale consists of five items. CTQ total scores can range from 25 to 125. The internal consistency of the CTQ in the current sample was high (α = 0.895).

Participants completed the *20-Item Toronto Alexithymia Scale* (TAS-20; German version: [54]) to assess alexithymia. This self-report questionnaire consists of 20 items and measures three core aspects of alexithymia: *difficulties in identifying feelings*,* difficulties in describing feelings*, and *externally oriented thinking* [55]. Total scores can range from 20 to 100. Scores from 52 to 60 are interpreted as indicating possible alexithymia, whereas scores of 61 or above are considered as indicating clinical levels of alexithymia [56]. The internal consistency of the TAS-20 in the current sample was very good (α = 0.903).

All participants also completed the state and trait versions of the *State-Trait Anxiety Inventory* (STAI; German version: [57]) to measure their current and dispositional anxiety. The STAI comprises two distinct versions, one for trait anxiety (STAI-T) and one for state anxiety. Each version contains 20 items. The total scores can range from 20 to 80. A higher score indicates a higher level of anxiety. In the present sample, the internal consistency of the STAI-T was excellent (α = 0.965).

Part B of the Trail Making Test (TMT-B; [58]) was administered as a measure of cognitive flexibility. This paper-pencil test requires participants to connect numbers and letters in ascending order while time is measured. The total time needed for completion of this task serves as an indicator of attention-switching control. Lower times indicate higher levels of performance.

### Eye tracking task

#### Stimuli and procedure

**Fig. 1:**
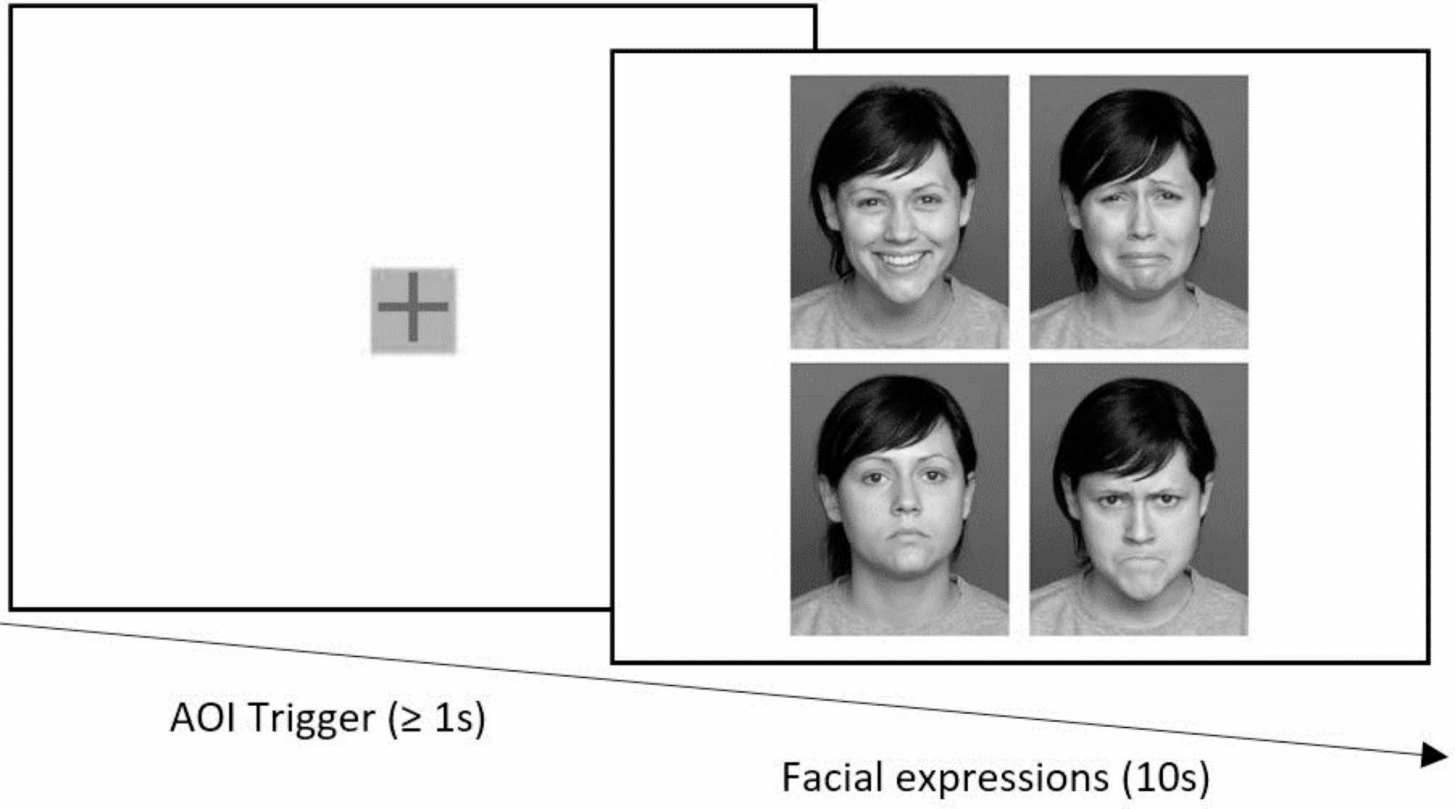
Example^2^ of an experimental trial (AOI = Area of Interest). The depicted model is 140_y_f from the MPI FACES database [55]

We administered a free-viewing paradigm to assess participant’s attention allocation to different facial expressions. This task has been previously applied by our research group to investigate depression-related attentional biases for emotional information [29, 47]. Stimuli consisted of 80 photographs of 20 actors (10 female), which were selected from the validated Lifespan Database of Adult Emotional Facial Stimuli [59].^1^ Four categories of facial expressions were used in our study: happy, angry, sad, and neutral. Each actor clearly expresses each of the emotional facial expressions. The facial expressions were arranged in a 2 × 2 matrix and presented simultaneously on a computer screen. The display size of each facial expression was 13 cm high and 11 cm wide. The images were presented in colour against a white background. Participants were instructed to view the presented photographs naturally. The instruction was given via the computer screen. A trial consisted of facial expressions of the same actor, and each actor was only presented once. Each trial started with a grey fixation cross, presented against a white background (see Fig. 1). The fixation cross was presented until a fixation of 1000 ms. Subsequently, the four facial expressions were presented for 10 s. The facial expressions were presented with equal frequency in each corner and appeared in their original colour against a white background. The experiment consisted of 20 trials with a total duration of approximately 4 min.

#### Apparatus

The stimuli were presented on a 22-inch widescreen monitor with a resolution of 1680 × 1050. Stimulus presentation and recording were executed using a SMI-customized Dell laptop (IView X laptop). Eye movements were recorded throughout the experiment. The recording was carried out using an IView X RED250 remote system manufactured by SensoMotoric Instruments (SMI). The IView X RED250 is a video-based eye-tracking device that has a sample frequency of 250 Hz and a gaze position accuracy of 0.4°. The eye-tracker compensates for movements; therefore, a chin rest is not required. SMI Experiment Center software was used for stimulus presentation and synchronization with recorded eye movements.

#### Eye movement parameter

The data was computed by a velocity-based algorithm with a minimum fixation duration of 100 ms, a minimum saccade duration of 22 ms, and a peak velocity threshold of 40°/s (see also: [47]). BeGaze 3.4.27 software was used to define areas of interest (AOIs) in each trial. The surface of the images of each facial expression category were defined as an AOI. The AOIs of all facial expression categories had the same size. The parameters *dwell time* and *entry time* were calculated. *Entry time* was used as an indicator for initial gaze orientation. It was defined as the time between stimulus onset and the first fixation on the AOI in milliseconds. It was calculated by averaging across participants for each AOI separately. Entry times lower than 20 ms were excluded from data analysis. Our threshold of 20 ms was set due to the observation that saccades have a mean duration of 43 ms with a minimum duration of 13 ms [60]. Furthermore, the saccadic reaction time of the human eye is considered to be 100 to 120 ms [61]. Therefore, in instances of small entry times, a saccade must have commenced prior to the stimulus onset. Consequently, we decided to exclude entry times lower than 20 ms.

*Dwell time* was used as an indicator for attention allocation, calculated by summing up the durations from all fixations and saccades, which hit the AOI in milliseconds. Therefore, *dwell time* refers to the duration of time that a participant’s gaze remains fixed within the boundaries of a specific AOI, taking into account attentional shifts. This means, if gaze shifts took place on a specific AOI but the gaze remained on the AOI the time associated with these shifts was included in the dwell time score. It was calculated by averaging the dwell time for each facial expression across trials and participants. As mentioned above, small entry times are not related to the stimulus onset due to the minimum duration of a saccade [60]and the saccadic reaction time [61]. Consequently, in instances with entry times lower than 20ms, the first fixation is not a reliable indicator of attentional processes. Because the parameter dwell time sums up the duration of all fixations in a certain AOI during stimulus presentation, the first fixation duration in cases of entry times smaller than 20 ms was not included in calculating the *dwell time*.

**Table 2:** Demographic and psychological characteristics of study groups

	BPD (*N* = 43)			NP (*N* = 43)				
	M	SD	Range	M	SD	Range		
Age	27.72	6.39	18–44	26.88	5.87	19–44		
School years	11.63	1.27	9–15	12.33	0.75	9–13	*	
TMT-B	64.81	23.22	33–132	57.30	15.81	34–102		
BDI-II	22.47	8.64	2–40	3.16	2.65	0–8	*	
STAI-T	59.79	7.88	41–77	33.86	7.41	22–57	*	
BSL-23	1.71	0.77	0.48–3.22	0.16	0.18	0-0.7	*	
TAS-20	54.84	11.97	30–79	37.86	10.06	23–69	*	
CTQ	62.65	17.25	35–113	31.86	5.80	25–50	*	

Note: BPD = borderline personality disorder; NP = non-patients; M = mean; SD = standard deviation; significant group differences at * *p* < .01; TMT-B = Trail Making Test Part B; BDI-II = Beck Depression Inventory-II; STAI-T = State-Trait Anxiety Inventory, trait version; BSL-23 = Borderline Symptom List; TAS-20 = 20-Item Toronto Alexithymia Scale; CTQ = Childhood Trauma Questionnaire

### General procedure

If study eligibility was granted by the SCID-I and SCID-II results, participants were scheduled for a second, experimental session. Prior to this session, participants were asked to complete a series of questionnaires, including the STAI-T, and the TAS-20. Participants were invited to the laboratory individually, gave informed consent, and received written instructions about the purpose of the experiment prior to its administration. Participants sat in front of a computer screen at a distance of approximately 70 cm. The experiment was conducted in a controlled environment, shielded from sunlight and with stable light from the ceiling. The lighting on the desk in front of the screen was approximately 570 lx, while the lighting at the position of the participant’s eyes was approximately 250 lx.^3^ Before starting the experiment, camera adjustments were made for the best capture. A nine-point grid was used for calibration purposes. Thereafter, a separate validation procedure was conducted. The maximum visual deviation was a 0.5° visual angle. Participants were instructed to minimize movements of the head and the body. After successful calibration, the free-viewing task started. Within the same experimental session, participants took part in two other eye-tracking experiments after the free-viewing task. After the eye-tracking experiments, the participants were asked to complete a series of questionnaires and neuropsychological instruments, including the STAI-S, BDI-II, BSL-23, CTQ, and the TMT-B.

### Statistical analysis

A 2 (group: BPD and NP) x 4 (emotional category: happiness, anger, sadness, and neutral expression) mixed model analysis of variance (ANOVA) was calculated to determine whether BPD patients differ from non-patients concerning *entry time* (early attention allocation), and *dwell time* (late attention allocation). If the sphericity assumption was violated, the Greenhouse-Geisser [62] correction was applied. Effect sizes are reported: Cohen´s *d* for *t*-tests and partial ƞ^2^ for ANOVAs. The statistical analysis was conducted using SPSS software (version 29). The alpha level was α = 0.05, if not otherwise specified. The *p-*levels are one-tailed for analyses with a priori directional predictions. In all other cases, *p*-levels are two-tailed. Entry time data on the different facial expressions was further analysed for group-independent differences. For this purpose, Bonferroni-corrected (α / 6 = 0.008) paired *t*-tests were performed examining the differences between entry times of each facial expression. To further investigate interaction effects, Bonferroni-corrected (α / 4 = 0.0125) pairwise comparisons were conducted. In cases where the assumption of normal distribution of residuals was violated, an additional non-parametric Mann-Whitney U test for independent samples was performed. We refrained from calculating ANCOVAs controlling the effect of depressive symptoms or anxiety since depression and anxiety are central to the concept of borderline personality disorder so that removing negative affect (by removing anxiety or depressive symptoms) means that the remaining group variance has poor construct validity for borderline personality disorder (see [63] for a discussion of use and misuse of ANCOVAs in psychopathology research).

A series of bivariate correlations were calculated between dwell time parameters and clinical questionnaires (STAI-T, BSL-23, CTQ, BDI-II, and TAS-20) for each group separately using Pearson’s correlation coefficient. In the case of non-normally distributed variables and outliers (defined by three times the interquartile range), Spearman rank correlation coefficient was used instead. To account for multiple testing, the level of significance was adjusted in accordance with Bonferroni (α / 5 = 0.010). We did not calculate correlations across groups because this way of proceeding (pooling of data from different samples) violates the basic assumption of sampling from one population, which underlies the use of correlation coefficients (see [64]).

To analyse the relationships between entry time and dwell time in our study groups, we conducted bivariate correlation analyses between these eye-tracking variables for each facial expression. Additionally, we examined whether the correlation coefficients differed between study groups (see for statistical details Additional Table 1). Our findings indicated significant correlations between entry and dwell times for angry, sad, and happy faces in the NP group and for sad faces in the BPD group. However, correlation coefficients did not differ significantly between groups for any of the facial expression conditions (see Additional Table 1).

## Results

### Demographic and psychological variables: between-group comparisons

The BPD group differed significantly from the NP group regarding reported levels of depression (BDI-II; *t*(49.81) = -14.01, *p* < .001, *d* = -3.02), trait anxiety (STAI-T; *t*(84) = -15.72, *p* < .001, *d* = -3.39), CTQ total scores (*t*(51.38) = -11.11, *p* < .001, *d* = -2.40), borderline specific symptoms, (BSL-23; *t*(46.46) = -12.81, *p* < .001, *d* = -2.76), and alexithymia (TAS-20; *t*(84) = -7.12, *p* < .001, *d* = -1.54). Groups did not differ in cognitive flexibility (TMT-B; *t*(74.02) = -1.76, *p* = .084) but differed in number of years spent in school (*t*(76.87) = 3.10, *p* < .001). To further clarify the impact of school years on eye-movement behaviour, a series of bivariate correlations was conducted. Results indicated that the number of school years did not correlate with dwell time (all *p*s > 0.17) or entry time (all *p*s > 0.46) for any facial expression. This indicates that years spent in school is not related to the analysed eye-tracking parameters.

### Late attention allocation: between-group comparison of dwell times and relations with psychological variables

**Fig. 2:**
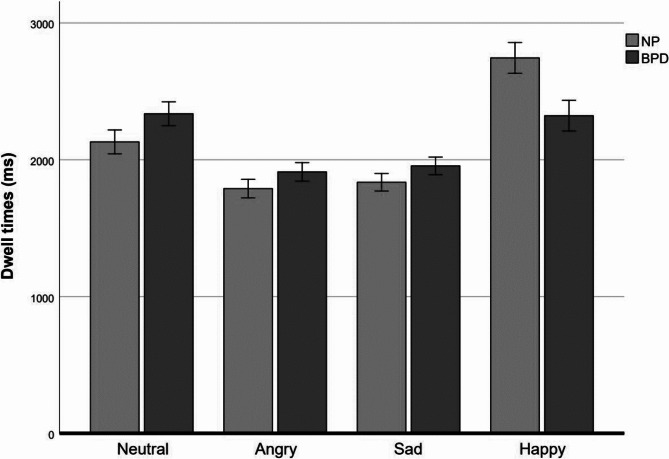
Dwell times in milliseconds (ms) for neutral, angry, sad and happy facial expression. Individuals with Borderline personality disorder (BPD) and non-patients (NP) are compared. Error bars represent standard error

Analysis of the dwell time data (see Fig. 2) revealed a significant main effect of facial expression category, *F*(2.04, 171.32) = 22.43, *p* < .001, partial ƞ^*2*^ = 0.21, and a significant interaction between facial expression category and group, *F*(2.04, 171.32) = 4.57, *p* = .011, partial ƞ^*2*^ = 0.05. Bonferroni-corrected (α = 0.0125) pairwise comparisons revealed that dwell time on happy faces was significantly shorter for the BPD group compared to the NP group (*t*(84) = 2.66, *p* = .005, *d* = 0.57). Dwell times on neutral faces (*t*(84) = -1.67, *p* = .049) were longer for the BPD compared to the control group but the difference failed to reach significance. Dwell times on sad (*t*(84) = -1.32, *p* = .095) and angry faces (*t*(84) = -1.28, *p* = .103) were not significantly different between groups. No result of the non-parametric test differed from the original post hoc independent *t*-tests. Therefore, only these are reported. Analysis of dwell time data comparing patients with and those without antidepressant medication revealed no group differences for any of the facial expressions (all *p*s > 0.14).

A post-hoc power estimate was performed using G*Power 3.1 software [65]. The results indicated an estimated statistical power of 1-β = 0.87 for the interaction effect on dwell time data.

Bivariate correlation analyses between dwell times and questionnaire data (BSL-23, STAI-T, BDI-II, TAS-20, CTQ) showed no significant results in the BPD group (all *p*s > 0.05, two-tailed). In the NP group, the bivariate correlations (Spearman rank) between dwell time on neutral faces and STAI-T (*r* = .383, *p =* .011, two-tailed), BSL-23 (*r* = .336; *p* = .028, two-tailed), and CTQ (*r* = .324; *p* = .034, two-tailed) failed to reach significance due to the Bonferroni-correction of the alpha level (α = 0.01). All other bivariate correlations between dwell times and questionnaire data were not significant in the NP group (all *p*s > 0.10, two-tailed).

### Early attention allocation: between-group comparison on entry times and analysis of expression condition

**Fig. 3:**
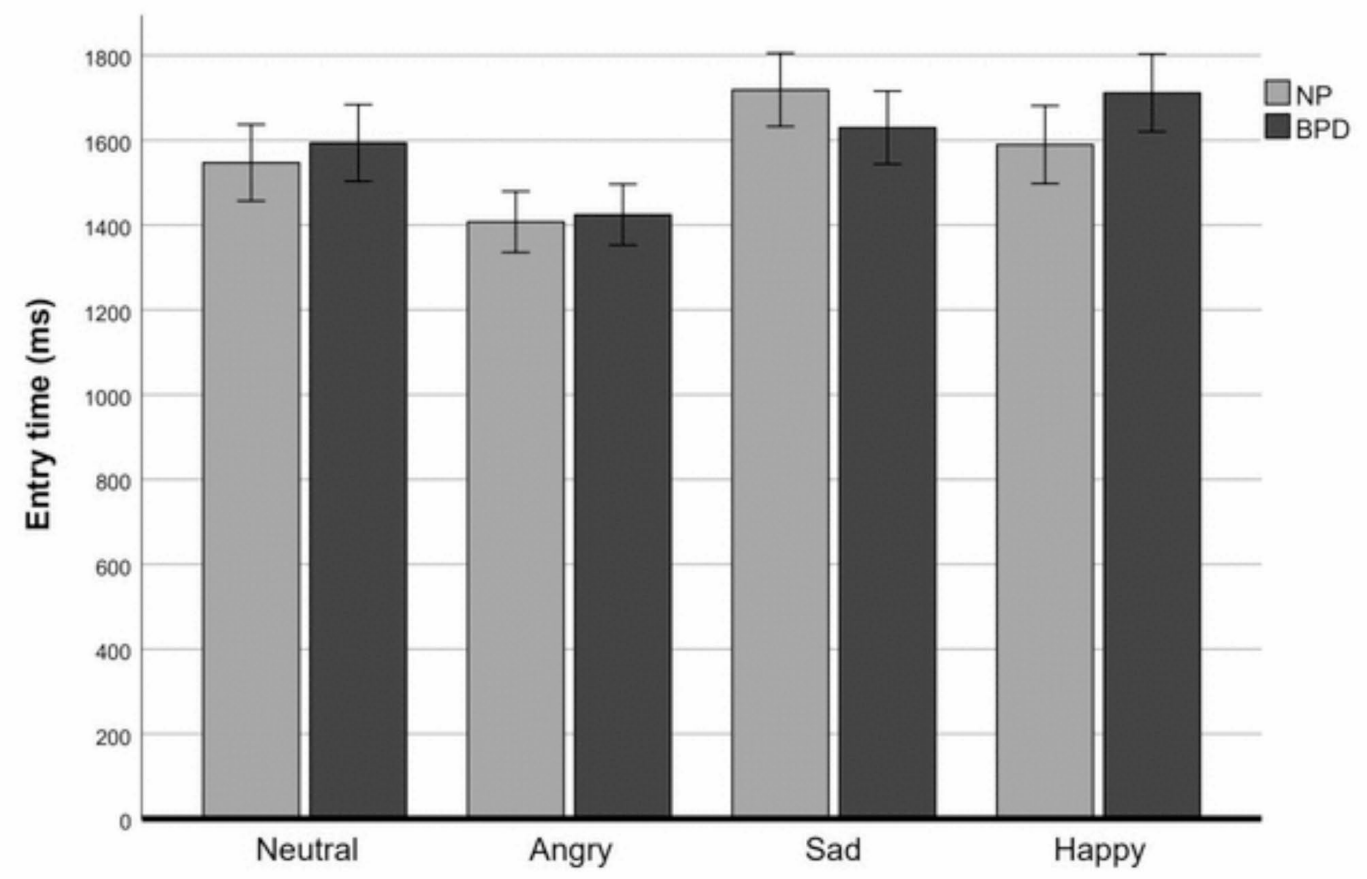
Entry times in milliseconds (ms) for neutral, angry, sad and happy facial expression. Individuals with Borderline personality disorder (BPD) and non-patients (NP) are compared. Error bars represent standard error

Descriptive statistics of entry time data for the BPD and the NP group are presented in Table 3; Fig. 3. Analysis of the entry time data revealed a significant main effect of facial expression category (*F*(3, 252) = 21.48, *p* < .001, partial ƞ^*2*^ = 0.20) and a significant group x facial expression category interaction (*F*(3, 252) = 3.02, *p* = .030, partial ƞ^*2*^ = 0.035). However, Bonferroni-corrected pairwise comparisons revealed no significant group differences in entry times for happy (*t*(84) = -0.94, *p* = .175), sad (*t*(84) = 0.73, *p* = .23), neutral (*t*(84) = 0.36, *p* = .358) or angry faces (*t*(84) = -0.17, *p* = .435). Again, no result of the non-parametric tests differed from the original post hoc independent *t*-tests. Analysis of entry time data comparing patients with and those without antidepressant medication revealed no group differences for any of the facial expressions (*p*s > 0.43). Collapsing entry times of both groups, Bonferroni-corrected paired *t*-tests indicated that entry time on angry facial expressions was significantly shorter compared to entry time on neutral facial expressions (*t*(85) = -4.23, *p* < .001 (two-tailed), *d* = − 0.46), happy facial expressions (*t*(85) = -6.53, *p* < .001 (two-tailed), *d* = − 0.70), and sad facial expressions (*t*(85) = -6.62, *p* < .001 (two-tailed), *d* = − 0.71). Additionally, entry time on neutral facial expressions was significantly shorter compared to sad facial expressions (*t*(85) = -3.17, *p* = .002 (two-tailed), *d* = − 0.34). There was no significant difference between the entry times on neutral facial expressions and happy facial expressions (*t*(85) = -2.36, *p* = .021, two-tailed) and no significant difference between the entry times on happy facial expressions and sad facial expressions (*t*(85) = -0.63, *p* = .529, two-tailed).

**Table 3:** Descriptive statistics of entry time data for study groups (entry time in milliseconds)

Emotion	BPD (*N* = 43)			NP (*N* = 43)			
condition	M	SD	Range	M	SD	Range	
Happy Sad Angry Neutral	1711 1630 1424 1594	646 592 487 626	734–3545 856–3609 602–3017 766–3653	1589 1719 1407 1547	554 539 457 557	890–3528 955–3061 739–3164 820–3725	

Note: BPD = Borderline personality disorder; NP = non-patients; M = mean; SD = standard deviation

## Discussion

The aim of this study was to investigate whether patients with BPD differ from NPs in their early and late attentional allocation to social emotional information. We assessed participants’ borderline symptoms as well as their trait anxiety, depressive symptoms, alexithymia, and experiences of childhood maltreatment, which can affect emotion perception. To the best of our knowledge, this is the first eye-tracking study examining early and late attention allocation to facial expressions in BPD using a multiple-stimulus free-viewing paradigm. The application of a free-viewing task allowed us to investigate self-generated gaze behaviour.

It was assumed that attention allocation in BPD is characterized by an *early threat vigilance* and a decreased *positive attentional bias*. We additionally assumed to find indications for *late threat avoidance*. Our results indicated that patients with BPD did not differ from NPs regarding early attention allocation, which contradicts the hypothesis of *early threat vigilance* in BPD. Regarding late attention allocation, our findings suggest that patients with BPD spent less time looking at happy faces than NPs, which confirms our hypothesis of decreased *positive attentional bias* in BPD. No significant differences were observed between the two groups for dwell times on negative facial expressions. Hence, we found no indications for *late threat avoidance* in BPD.

The main finding of this study is that BPD appears to be characterized by a decreased positivity bias, a reduced attentional preference for positive over negative and neutral information. Guiding attention toward positive stimuli increases reward perception and can have mood-enhancing effects [31]. The present results are in line with those of Bortolla et al. [19] who found that patients with BPD spent less time exploring positive socio-emotional content than NPs. Nevertheless, the findings of our study diverge from those of Bortolla et al. [28], which indicate that patients with BPD spent an equal amount of time exploring positive socio-emotional pictures as NPs. It is noteworthy that Bortolla et al.‘s study [28] observed a trend for a decreased attentional preference to positive socio-emotional content when pairwise-comparing the 18s stimulus condition between groups. Consequently, the results obtained in our study converge at least in part with those of Bortolla et al. [28] for long stimulus presentations. Therefore, long stimulus presentations may be necessary to detect a decreased positive attentional bias. An important methodological difference between Bortolla et al.’s studies and our investigation concerns the task administered. Bortolla et al. [19, 28] asked participants to rate the images immediately after their presentation while our participants looked freely at facial expressions. Free-viewing has been shown to be related to dorsolateral prefrontal cortical activity and is thought to reflect attentional responses that are indicative of more endogenous control in comparison with attention measures derived from task-related gaze behaviour [36]. The perception processes in recognition tasks seem to involve more feature-based, stimulus-driven visual processing. Our dwell time data suggest that BPD patients could be characterized by impairments in processes of spontaneous attention allocation, which appear, at least in part, under endogenous control. However, considering the findings of Bortolla et al.’s study [19] and our investigation it can be concluded that BPD patients manifest reduced sustained attention to positive contents in tasks assessing more stimulus-driven processes as well as in tasks measuring primarily self-generated processes. A decreased positive attentional bias may favour the occurrence of negative affects in BPD. Attention to positive emotions can modulate emotions via emotional contagion [66]. Subsequently, decreased attention to positive emotions may increase the perception of distress. Since BPD is associated with a low stress tolerance [3], decreased attention allocation to positive stimuli could play an important role in emotion dysregulation in BPD.

It must be acknowledged that the high rate of comorbid axis I disorders in the BPD group does not allow a strict attribution of the decreased positive attentional bias to BPD. Interestingly, there were no correlations between dwell time data and the levels of anxiety, depression, CM, borderline symptom severity or alexithymia in either of the groups. This indicates that self-generated attention deployment has no relationship with any of these clinical measures *within* these groups. However, this must be interpreted with caution due to the small sample size when calculating correlations for both groups individually. Interestingly, there is evidence from recent eye-tracking research using mood-induction that among NPs positively biased attention allocation is a stable, trait-like feature, rather than a state-like mood-dependent one [67].

Concerning late attention allocation towards negative facial expressions, we found no indications for *late threat avoidance*. This finding contradicts previous eye-tracking research from Bortolla et al. [19, 28], indicating that BPD patients explore negative socio-emotional content to a lesser extent than healthy individuals. These discrepancies may be attributed to the different stimuli or paradigm employed in Bortolla et al.‘s studies and those employed in the present study. The influential biosocial model of BPD states that patients with BPD are characterized by emotional (hyper-) sensitivity [3] and therefore might show altered attentional allocation towards threat. Furthermore, threat avoidance is commonly assumed in BPD (e.g [4, 35]). Nevertheless, we found no indications for threat avoidance in late processes of attentional allocation. Our results must be interpreted with caution for several reasons. The multi-stimulus free-viewing paradigm does not allow a strict differentiation between attentional approach and avoidance of facial stimuli. Since four facial expressions were presented simultaneously, dwell times may reflect approach tendencies towards a specific type of facial expression or avoidance of other facial expressions. Thus, we had no unambiguous measure of threat avoidance. It can also be argued that a threat needs to be self-relevant in order to elicit avoidance [68]. Further research (using for example pairs of faces combining a neutral face with a threatening one) is needed to clarify threat avoidance in BPD.

This study found no evidence for *early threat vigilance* in BPD. Our results are consistent with those of Bortolla et al. [19], who observed no differences between patients with BPD and healthy controls in early attention allocation. However, Bortolla et al. [28] observed an increased first fixation latency for negative emotional content, which contradicts the results of our study. Since previous research provided empirical evidence for faster attentional orientation towards threat [25–27] and theoretical models predict early threat vigilance in BPD [3], the inconsistencies need to be carefully discussed. It has to be noted that entry times were shortest for angry facial expressions, indicating a group-independent threat vigilance in our sample. This finding is in accordance with evidence from research on attention and facial emotion [69], which indicates that angry faces preferentially capture attention at an early level of processing in healthy individuals when they are presented simultaneously with other facial expressions. The fact that we found an early vigilance effect when collapsing data of both groups shows that the multiple-stimulus free-viewing paradigm with facial expressions could be suitable for research on early attention processes and in particular early threat vigilance. However, we found no difference between the BPD and the NP group. It is important to note that the eye-tracking studies reporting early vigilance [25–27] or early avoidance [28] in BPD used different experimental paradigms and stimuli, which can have an impact on early attention allocation [21]. Therefore, the inconsistencies with previous findings may reflect differences in the experimental paradigms. It is possible that *early threat vigilance* in BPD can only be detected when facial expressions are presented individually, and gaze behaviour is analysed specifically in relation to the eye region. Individually presented facial expressions may represent more salient threat stimuli, which could therefore capture early vigilance towards threat in BPD, as observed in studies using the emotion recognition paradigm [25–27].

As eye-tracking behaviour in experimental settings can reflect eye-movements towards faces as they occur in real world situations [70], our findings may contribute to a better understanding of attention allocation in BPD. The findings may have therapeutic implications for the treatment of BPD patients. An attentional bias modification training could be helpful to promote gaze behaviour towards positive social expressions [71].

There are several limitations to this study that need to be acknowledged. Although we found no statistically significant difference in school years between groups and no significant correlation of school years with eye-tracking data, groups were not matched for this variable. Moreover, the common issue of comorbidity in BPD research was not fully addressed. The BPD group was characterised by a high proportion of affective and anxiety disorders, which did not allow subgrouping of BPD with or without comorbid axis I disorders. Differences in the composition of comorbid axis I disorder may decrease comparability between studies. Future research based on larger samples could investigate how axis I comorbidity affects attention allocation to emotional content, e.g., by comparing groups with no comorbid disorder, comorbid clinical depression or anxiety disorder. A further limitation of our study is that the reliability of the early attention allocation parameter (i.e., entry time) appears low in free-viewing paradigms [72]. Finally, future research should include measures of emotion regulation to further our understanding of the mechanisms involved in attention allocation to emotions in BPD.

## Conclusions

This eye-tracking study aimed to examine early and late attention allocation towards emotional facial expressions in BPD using a multi-stimulus free-viewing paradigm. Free-viewing tasks allow to investigate self-generated attention processes. Patients with BPD were found to exhibit a decreased positive attentional bias compared to NPs at a late processing stage. Thus, BPD patients showed reduced attentional preference for happy facial expressions. Decreased preference of positive stimuli could be part of emotion regulation impairments and add to the vulnerability for negative affects in BPD. According to our results, BPD patients seem not to be characterised by early threat vigilance or late threat avoidance.

## Electronic supplementary material

Below is the link to the electronic supplementary material.

Supplementary Material 1

## Data Availability

The datasets used and analysed during the current study are available from the corresponding author upon reasonable request.

## Footnotes

1 Stimuli included pictures from the following actors: 006, 008, 010, 014, 020, 022, 028, 029, 031, 032, 035, 038, 041, 043, 045, 048, 050, 056, 057, and 062.

2 The pictures shown are representative for the stimulus material but were not used in the present study.

3 Lighting was measured using the Mavolux 5032B luxmeter (Gossen, Nuremberg, Germany).

## Abbreviations

AOI: Area of interest
ANOVA: Analysis of Variance
ANCOVA: Analysis of Covariance
BDI-II: Beck Depression Inventory
BPD: Borderline personality disorder
BSL-23: Borderline Symptom List
CM: Childhood maltreatment
CTQ: Childhood Trauma Questionnaire
NP: Non-patient
STAI: State-Trait Anxiety Inventory
STAI-S: State-Trait Anxiety Inventory, State-Version
STAI-T: State-Trait Anxiety Inventory, Trait-Version
SCID-I and II: Structured Clinical Interview for DSM-IV (axis I disorders, axis II disorders)
TAS-20: 20-Item Toronto Alexithymia Scale
TMT-B: Part B of the Trail Making Test
