# Supplementary material for: Reduced positive attentional bias in patients with borderline personality disorder compared with non-patients: results from a free-viewing eye-tracking study
Source: Borderline Personal Disord Emot Dysregul. 2024 Sep 16;11:24. doi: 10.1186/s40479-024-00267-y (PMC11403868; doi:10.1186/s40479-024-00267-y)
Supplement: Supplementary file 1 — Supplementary Material 1 [file 40479_2024_267_MOESM1_ESM.docx]

| **Additional Table 1.** Correlations between entry and dwell times for the emotion face conditions in the BPD and the NP group and comparison of correlations based on *Z* statistics (following the calculation of Eid et al. (2011) for independent samples). | | | | | |
| --- | --- | --- | --- | --- | --- |
|  | BPD group | | NP group | |  |
| Correlation of entry time with dwell time | *r* | Sig. | *r* | Sig. | Difference between groups (Sig.)^a^ |
| Neutral | -.248^b^ | .109 | .039^b^ | .805 | .096 |
| Angry | .152 | .330 | .345* | .024 | .178 |
| Sad | .339*^b^ | .026 | .474** | .001 | .234 |
| Happy | -.059^b^ | .708 | -.302*^b^ | .049 | .129 |
| *Note*: significant correlations at * *p* < .05 (two-tailed) and ** *p* < .01 (two tailed).  ^a^ Calculated using Lenhard and Lenhard´s (2014) online calculator for correlations.  ^b^ The Spearman rank correlation coefficient is reported due to the presence of non-normally distributed variables containing outliers (three times interquartile range). | | | | | |

*References:*

Eid, M., Gollwitzer, M., & Schmitt, M. (2011). Statistik und Forschungsmethoden Lehrbuch. Weinheim: Beltz.

Lenhard, W. & Lenhard, A. (2014). Signifikanztests bei Korrelationen. verfügbar unter: https://www.psychometrica.de/korrelation.html. Psychometrica. DOI: 10.13140/RG.2.1.2954.1367
